# Radiocarbon dates and stable isotope data for human and animal skeletal remains from a passage grave in Kierzkowo, Poland (Late Neolithic and modern period)

**DOI:** 10.1016/j.dib.2022.108011

**Published:** 2022-03-03

**Authors:** Łukasz Pospieszny, Szymon Nowaczyk

**Affiliations:** a Polish Academy of Sciences, Institute of Archaeology and Ethnology, Rubież 46, Poznań 61-612, Poland; b School of Earth Sciences, University of Bristol, Wills Memorial Building, Queens Road, Bristol BS8 1RJ, United Kingdom; c Archaeological Museum in Biskupin, Biskupin 17, Gąsawa 88-410, Poland

**Keywords:** ^14^C dating, Carbon and nitrogen stable isotope analyses, Megalithic tomb, Globular Amphora culture

## Abstract

This dataset is comprised of paired radiocarbon (^14^C) dates, and carbon (*δ*^13^C) and nitrogen (*δ*^15^N) stable isotope ratios received for skeletal remains of 18 humans and 6 animals. These remains were archaeologically-derived from a Late Neolithic passage grave in Kierzkowo, located in today's north-central Poland. All human individuals were sexed and aged by physical anthropologists; animal skeletal remains were identified by zooarchaeologists. Collagen samples were extracted from bones, radiocarbon dated by accelerator mass spectrometry (AMS) and stable carbon and nitrogen isotope compositions measured by isotope ratio mass spectrometry (IRMS). The samples were collected and analysed to establish the absolute chronology of the tomb, estimate the frequency of burials, reconstruct the diets of humans and animals and trace their temporal changes. This is a largest dataset for skeletal samples (*n* = 24) from a single megalithic tomb in East-Central Europe and has a utility to be reused in various archaeological and palaeoenvironmental studies.

## Specifications Table

**Table:** 

Subject	Archaeology
Specific subject area	AMS radiocarbon dating Stable isotope analyses Bioarchaeology
Type of data	Table Figure
How the data were acquired	For measuring nitrogen and carbon contents in whole bone: Thermo Flash EA 1112 Series elemental analyser. For radiocarbon dating: Compact Carbon AMS spectrometer. For carbon and nitrogen stable isotope analyses: Thermo Flash EA 1112HT elemental analyser coupled with a Thermo Delta V Advantage isotope ratio mass spectrometer.
Data format	Raw
Parameters for data collection	All data passed the established collagen quality control (QC) criteria: collagen yield (%Col) greater than 0.5% and lower than 22% by mass of sample, and atomic carbon-to-nitrogen ratio (C/N) between 2.9 and 3.6 [1].
Description of data collection	Human and animal skeletal remains came from archaeological excavations of a Late Neolithic megalithic tomb in Kierzkowo, Poland. Bulk bone collagen was extracted from 24 bones and bone fragments. Coupled radiocarbon dating and carbon and nitrogen stable isotope analyses were conducted for all samples. One human individual was sampled twice.
Data source location	Institution: Institute of Archaeology and Ethnology, Polish Academy of Sciences City: Poznań Country: Poland
Data accessibility	The dataset is available at a public repository IsoArcH, an open access isotope web-database for bioarchaeological samples. Repository name: IsoArcH (www.isoarch.eu) [2] Data identification number: 10.48530/isoarch.2021.013 Direct URL to data: https://doi.isoarch.eu/doi/2021.013[3] Data is available under the Creative Commons BY-NC-SA 4.0 license.

## Value of the Data

• Archaeologists can use this dataset for studying the appearance and development of the megalithic architecture in East-Central Europe.
• Historians can benefit from the radiocarbon evidence of previously undetected reuse of the monument in the modern period.
• This dataset is useful for reconstructing human and animal diets and tracing their changes over time with high temporal resolution enabled by direct ^14^C dating of each isotopically-studied bone collagen sample.
• *δ*^13^C and *δ*^15^N stable isotope data have utility for reconstructing food webs, trophic shifts, animal husbandry practices and vegetation cover in the Late Neolithic.
• ^14^C dates can be used for building absolute chronologies and modelling past demographic changes.

## 1 Data Description

**Fig. 1:**
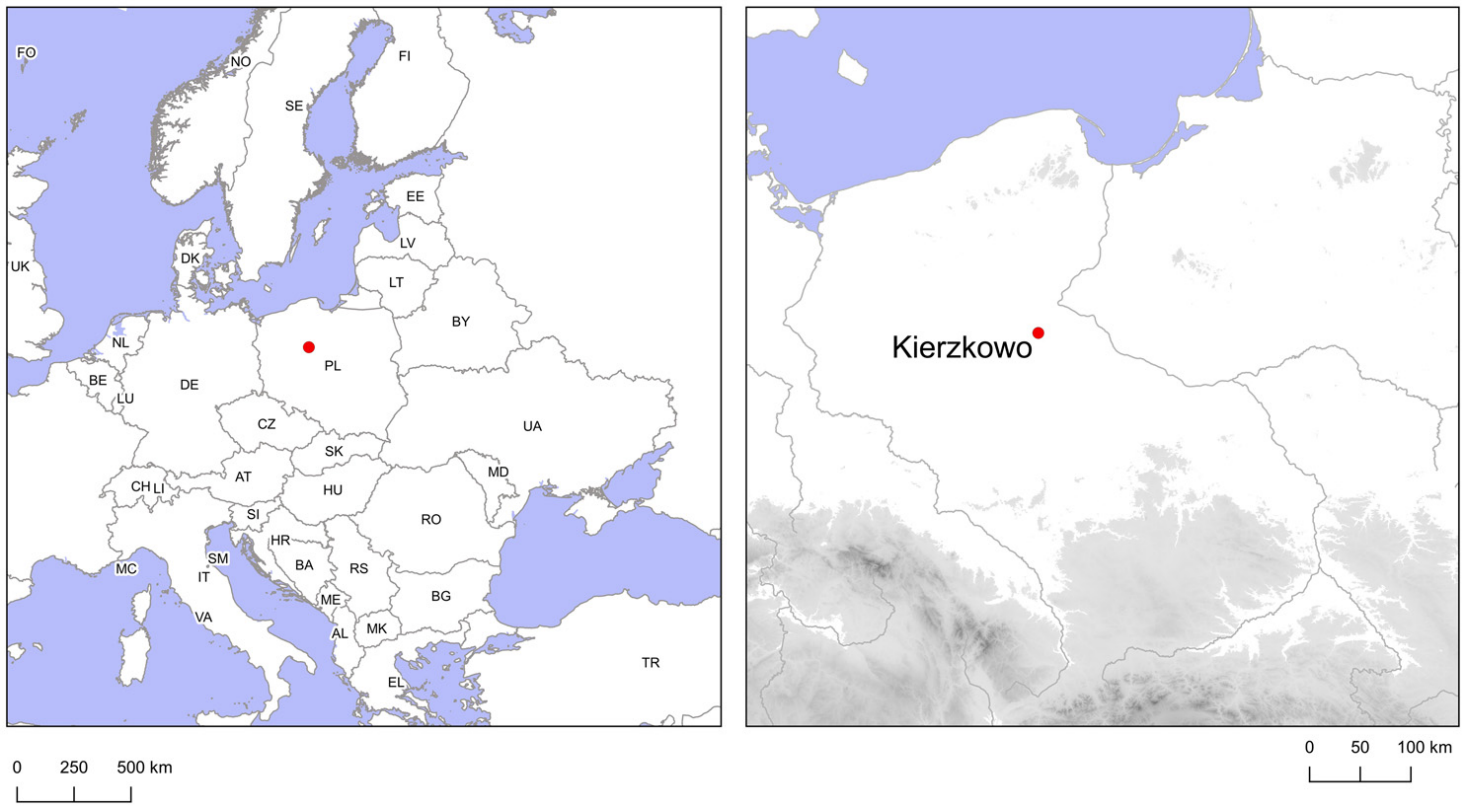
Map showing the location of the passage grave in Kierzkowo, covered by this dataset.

This dataset consists of AMS radiocarbon (^14^C) dates, and carbon and nitrogen stable isotope ratios (*δ*^13^C and *δ*^15^N values, respectively) of bulk collagen extracted from 18 human and 6 animal skeletons from a Late Neolithic passage grave from Kierzkowo, site 13, north-central Poland (Fig. 1). The tomb was excavated from 1983 to 1984 and initially dated with the use of conventional radiocarbon methods [4]. Archived materials and documentation were revisited in 2016 [5]. Sampled human remains were found both inside and outside the burial chamber, faunal samples represented only the remains unearthed outside the chamber. Samples were collected by the authors and submitted for analyses at the Poznań Radiocarbon Laboratory and at the Institute of Geological Sciences of the Polish Academy of Sciences (both in Poland). Independently, ancient DNA (aDNA) analyses, supported by AMS radiocarbon dating at the Beta Analytic in Miami (USA), were conducted at the University of Florence (Italy) [6].

**Fig. 2:**
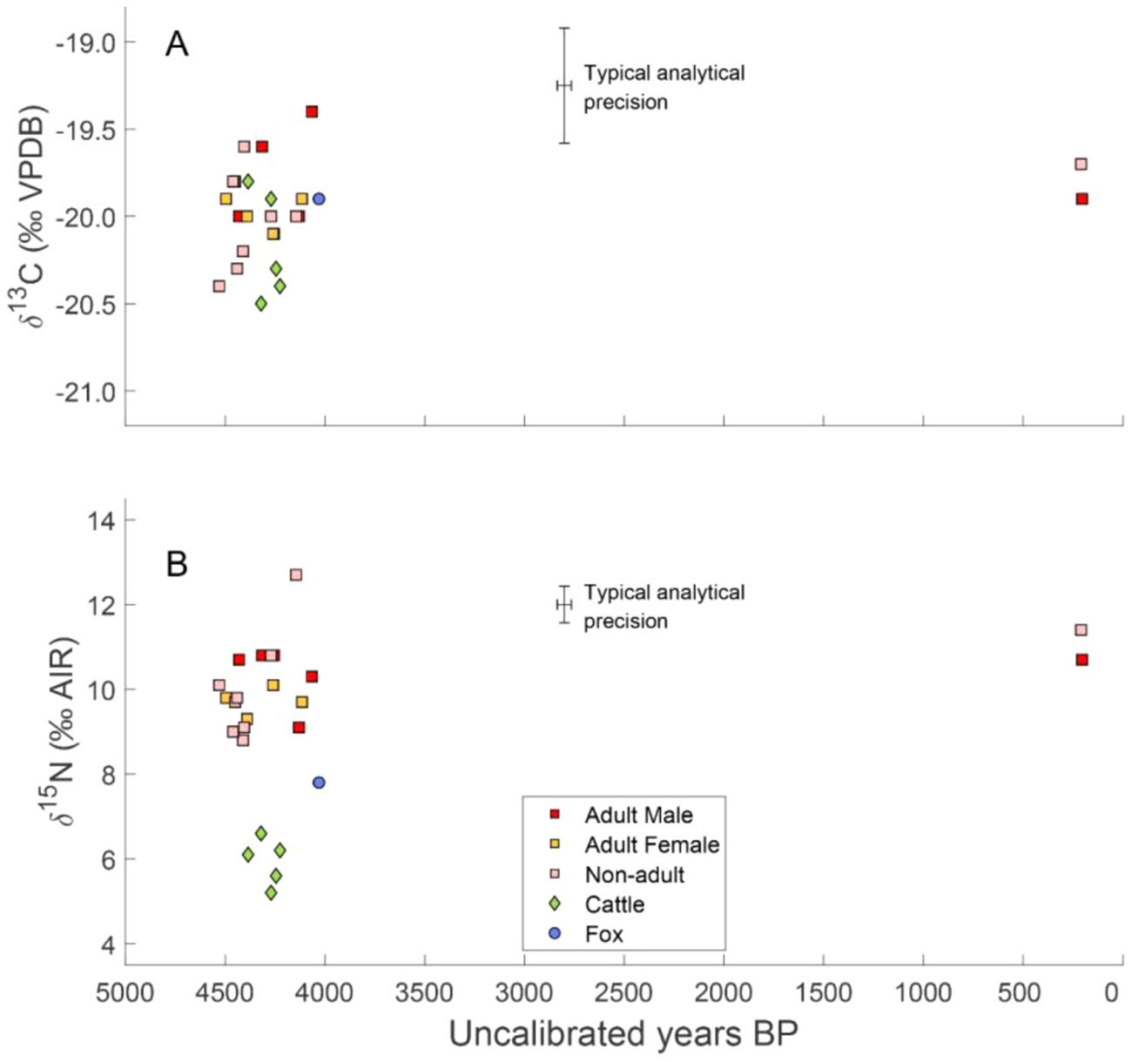
(A) *δ*^13^C and (B) *δ*^15^N values for humans and animals from Kierzkowo against their radiocarbon ages.

Inside the collective tomb the skeletal remains of 27–29 people were discovered, mostly disarticulated and intermingled, which hindered the estimation of the minimum number of individuals. Skeletal remains of domesticated and wild animals were found inside the burial chamber, occasionally mixed with human bones, and also outside the chamber, often articulated (remains of carcasses). The monument was built by the people of the Globular Amphora culture (GAC), and used for human burials and animal offerings between approx. 3180 and 2760 BC, i.e., in the Late Neolithic (ca. 3450-2750 BC). It was re-used as a graveyard in the modern period, between 17th and 18th c. AD (Fig. 2).

**Fig. 3:**
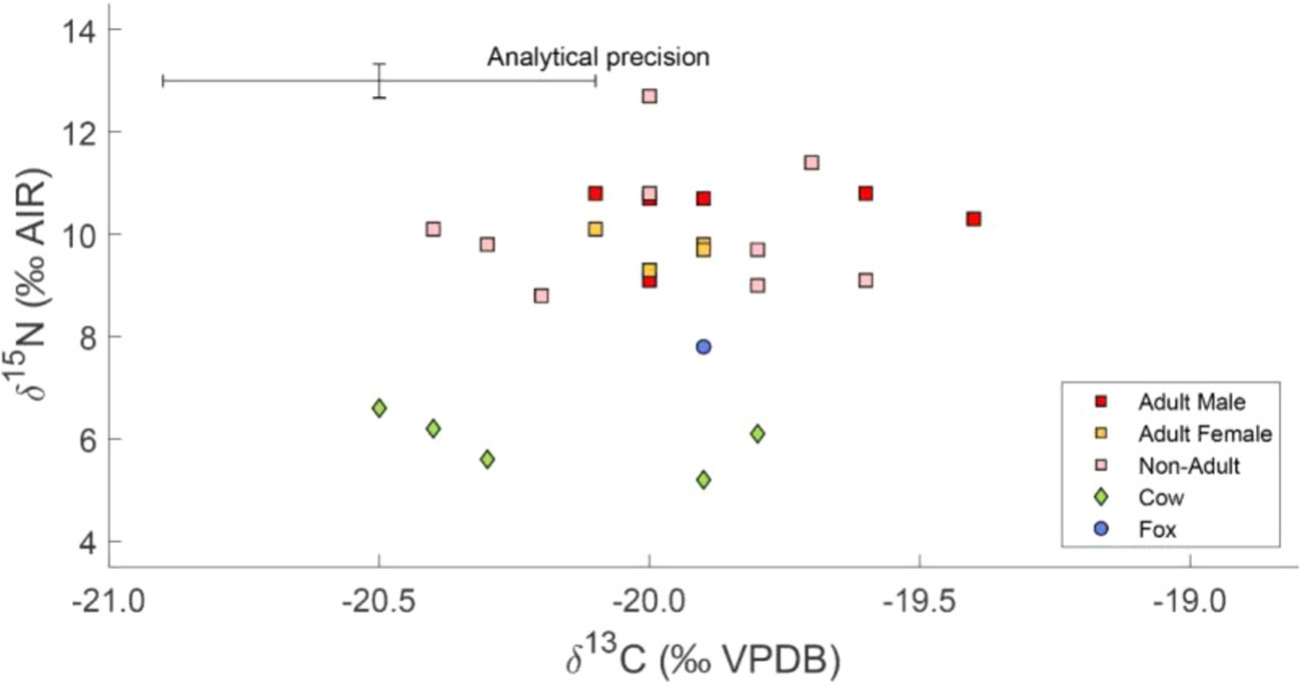
*δ*^13^C and *δ*^15^N values for humans and animals from Kierzkowo.

Table 1 presents individual data including species, skeletal element sampled, AMS ^14^C dates, *δ*^13^C and *δ*^15^N values (Fig. 3), and collagen preservation quality indicators such as collagen yield, atomic concentration of carbon and nitrogen, and atomic carbon-to-nitrogen ratio. For humans also age at death and sex are provided. Summary of isotopic values for humans is given in Table 2.

**Table 1:** Samples characteristics, AMS radiocarbon ages, and isotopic data with quality control information for humans and animals from Kierzkowo.

Individual Reference	Species	Sampled skeletal part	Age at death (years)	Sex	^14^C sample ID	^14^C uncalibrated Age (BP)	*δ*^13^C (‰VPDB)	*δ*^15^N (‰AIR)	%Col	%C	%N	C:N_atomic_
1	human	tarsal	18–19		Poz-86572	4270±35	–20	10.8	5.6	46.5	16.6	3.3
2	human	cranium	20–50	M	Poz-86784	4315±35	–19.6	10.8	9.7	42.7	15.4	3.2
3	human	tibia	20–35	M	Poz-86569	4430±30	–20	10.7	1.3	40.4	14.3	3.3
4	human	scapula	30–40	F	Poz-86570	4495±35	–19.9	9.8	7.4	45.2	16.1	3.3
5	human	scapula	7–14		Poz-86568	4530±35	–20.4	10.1	7	48.8	17.6	3.2
6	human	tibia	14		Poz-86565	4450±35	–19.8	9.7	3.3	44.5	16.1	3.2
7	human	scapula	7–8	M	Poz-86564	4410±35	–20.2	8.8	6.2	48.1	17.6	3.2
8	human	scapula	2–3		Poz-86563	4145±35	–20	12.7	9.3	47.9	17.1	3.3
9	human	radius	10–14	M	Poz-86566	4460±35	–19.8	9	6.7	45	16.2	3.2
10	human	cranium	20–30	F	Poz-86780	4115±35	–19.9	9.7	8.2	40.4	13	3.6
11	human	mandible	20–30	M	Poz-86497	4130±30	–20	9.1	1.8	44.2	15.6	3.3
11	human	vertebrae	20–30	M	Poz-86573	4255±30	–20.1	10.8	3.2	44.3	15.7	3.3
12	human	cranium	40–50	F	Poz-86574	4390±35	–20	9.3	1	41.7	14.8	3.3
13	human	cranium	30–50	F	Poz-86498	4260±35	–20.1	10.1	1.4	34.8	12.4	3.3
14	human	scapula	4–5		Poz-86567	4440±35	–20.3	9.8	7	43.6	15.4	3.3
15	human	mandible	12–14		Poz-86782	4405±35	–19.6	9.1	6.5	45.1	16.1	3.3
16	human	mandible	30–40?	M	Poz-86783	4065±35	–19.4	10.3	2.8	43.9	15.6	3.3
17	human	tibia	20–30	M	Poz-86560	205±30	–19.9	10.7	16.8	46.8	16.7	3.3
18	human	cranium	5–7		Poz-86562	210±30	–19.7	11.4	9.7	48.6	17.4	3.3
19	cattle	mandible			Poz-86575	4385±35	–19.8	6.1	4.2	45.1	15.8	3.3
20	cattle	mandible			Poz-86577	4270±35	–19.9	5.2	2.5	47.9	17.2	3.2
21	cattle	2nd molar			Poz-86578	4245±35	–20.3	5.6	3.6	48	16.8	3.3
22	cattle	mandible			Poz-86581	4225±35	–20.4	6.2	1.8	47	16.7	3.3
23	cattle	mandible			Poz-86576	4320±30	–20.5	6.6	4.8	49.1	17.5	3.3
24	fox	mandible			Poz-87638	4030±30	–19.9	7.8	5.9	48.7	17	3.3

Abbreviations: F = female; M = male.

**Table 2:** Descriptive statistics of isotopic data for humans from Kierzkowo.

		*δ*^13^C (‰ VPDB)				*δ*^15^N (‰ AIR)			
Category	*n*	mean	sd	min	max	mean	sd	min	max
Adult males	6	–20.0	0.2	–20.1	–19.4	10.0	1.4	9.1	10.8
Adult females	4	–19.9	0.2	–20.1	–19.9	10.5	0.5	9.3	10.1
Non–adults	9	–19.9	0.3	–20.4	–19.6	10.1	0.8	8.8	12.7

Note that individual 11 (adult male) was analysed twice.

## 2 Experimental Design, Materials and Methods

Osteological analysis concluded that a minimum of 27 individuals were placed inside the tomb, with a maximum number of individuals determined to be 29. 19 bone samples, representing 18 of these individuals were taken for collagen extraction. Skeletal elements of known location and stratigraphic position (noted and marked on plan drawings) were prioritised. In other cases, homonymous bones or those clearly indicating the age of an individual at death were collected to avoid sampling the same individual twice. Only for one individual two different bones were selected, a femur and two thoracic vertebrae stuck together in sediment, a sole example of articulated bones within the tomb.

In case of animals, a single bone of a fox, and 5 bones of cattle were collected. For the latter 5 homonymous bones or those deriving from individuals with a clearly distinct age at the time of death were selected to ensure they represent distinct specimens.

Selected fragments of human and animal bones were submitted to the Poznań Radiocarbon Laboratory (in Poznań). AMS ^14^C dating of bones was performed on collagen extracted using procedures originally described by Longin [7], with further modifications [8,9].

Before extraction, degree of collagen degradation was checked by measuring content of nitrogen and carbon in raw bone, using Thermo Flash EA 1112 Series analyser. The samples regarded suitable for collagen extraction (with nitrogen content not lower than 0.6% by mass) were cleaned mechanically, ultrasonically washed in ultrapure water, crushed in a mill to granulation <0.3 mm, and the bone powder was treated with 2 M HCl (room temp., 20 min), and 0.1 M NaOH (room temp., 1 h). After each step of treatment, the sample was centrifuged, and the residuum was collected. Extraction of collagen was processed in HCl (pH = 3, 80 °C, 10 h), and after centrifugation, residuum was removed. The extracted collagen was then ultrafiltered on pre-cleaned Vivaspin 15 MWCO 30 kD filters [9]. Aliquots of collagen were then combusted in closed (sealed under vacuum) quartz tubes, together with CuO and Ag wool, in 900 °C through 10 h. The obtained gas (CO_2_ + water vapour) was dried in a vacuum line, and reduced with hydrogen (H_2_), using 2 mg of Fe powder as a catalyst [10]. The obtained mixture of carbon and iron was pressed into cathodes suitable to work in the ion source of AMS ^14^C spectrometer. Content of ^14^C in a sample of carbon was measured using the spectrometer “Compact Carbon AMS” [11], by comparing intensities of ion beams of ^14^C, ^13^C and ^12^C measured for each sample and for standard samples (modern standard: “Oxalic Acid II” and standard of ^14^C-free carbon: “background”). Conventional ^14^C ages were calculated using correction for isotopic fractionation [12], basing on ratio ^13^C/^12^C measured in the AMS spectrometer simultaneously with the ratio ^14^C/^12^C. The aliquots of collagen samples were submitted to the Isotope Dating and Environment Research Laboratory at the Institute of Geological Sciences of the Polish Academy of Sciences (in Warsaw) for carbon and nitrogen isotope measurements.

Stable isotopic composition of carbon (*δ*^13^C) and nitrogen (*δ*^15^N) was determined using a Thermo Flash EA 1112HT elemental analyser coupled with a Thermo Delta V Advantage isotope ratio mass spectrometer in a Continuous Flow system. Samples wrapped in tin capsules were combusted at 1020 °C, and released gases (CO_2_ and N_2_) separated in a GC column were transferred to MS source through a capillary. Isotope ratios were normalized to a calibration curve based on international standards USGS 40, USGS 41, IAEA 600, and expressed relative to VPDB for *δ*^13^C and to atmospheric nitrogen for *δ*^15^N, with uncertainties (1*σ*) of ±0.33‰ for carbon and ±0.43‰ for nitrogen isotope measurements.

## 3 Data Analysis

Radiocarbon dates received for human remains fall into two groups (Fig. 2). The first grouping includes 17 radiocarbon dates, which represent 16 of the 21–23 total individuals placed inside the chamber. They range from 4530 ± 35 BP to 4065 ± 35 BP and inform about the time-span and frequency of burials in the Late Neolithic. The other two dates, 210 ± 30 BP and 205 ± 30 BP, were received for 2 out of 6 skeletons discovered outside the chamber. They inform about the unexpected re-use of the tomb in the modern period. Skeletal remains of cattle and fox, discovered outside the chamber, yielded 6 dates ranging from 4385 ± 35 to 4030 ± 30 BP. AMS ^14^C dates must be calibrated using dedicated software [13] and northern hemisphere radiocarbon age calibration curve [14]. Isotope data was obtained for all 19 samples of human bones collected from 18 individuals, and all 6 samples of animal bones (Table 1). *δ*^13^C values for humans range from –20.4‰ to –19.4‰ (Table 2). *δ*^15^N values vary from 8.8‰ to 12.7‰ (Table 2). The highest *δ*^15^N ratio, 12.7‰, was received for the remains of a child who died at the age of 2–3 years and could be caused by breastfeeding [15]. For cattle, *δ*^13^C values range from –20.5‰ to –19.8‰, *δ*^15^N values vary from 5.2‰ to 6.6‰ (Fig. 3). In the case of the fox, these values are –19.9‰ and +7.8‰, respectively, and are intermediate between herbivores and humans.

## Ethics Statements

No data were collected from modern human populations or individuals with known relatives.

## CRediT authorship contribution statement

**Łukasz Pospieszny:** Conceptualization, Methodology, Data curation, Writing – original draft, Visualization, Project administration, Funding acquisition. **Szymon Nowaczyk:** Writing – review & editing, Project administration, Funding acquisition.

## Declaration of Competing Interest

The authors declare that they have no known competing financial interests or personal relationships that could have appeared to influence the work reported in this paper.

## References

[1] Guiry E.J., Szpak P. (2021). Improved quality control criteria for stable carbon and nitrogen isotope measurements of ancient bone collagen. J. Archaeol. Sci..

[2] Salesse K., Fernandes R., de Rochefort X., Brůžek J., Castex D., Dufour É. (2018). IsoArcH.eu: an open-access and collaborative isotope database for bioarchaeological samples from the Graeco-Roman world and its margins. J. Archaeol. Sci. Rep..

[3] Pospieszny Ł., Nowaczyk S. (2021). Radiocarbon dates and stable isotope data for human and animal skeletal remains from a passage grave in Kierzkowo. IsoArcH.

[4] Lisi C.S., Pessenda L.C.R., Cruz M.V.L., Pessin G., Pazdur M.F. (1996). Purificação de CO_2_ sintetizado de amostras de ossos destinadas à datação por ^14^C via espectrometria de cintilação líquida. Quím. Nova.

[5] Nowaczyk S., Pospieszny Ł., Sobkowiak-Tabaka I. (2017). Megalityczny grobowiec kultury amfor kulistych z Kierzkowa na Pałukach: milczący świadek kultu przodków z epoki kamienia.

[6] Vai S., Diroma M.A., Cannariato C., Budnik A., Lari M., Caramelli D., Pilli E. (2021). How a paleogenomic approach can provide details on bioarchaeological reconstruction: a case study from the Globular Amphorae culture. Genes.

[7] Longin R. (1971). New method of collagen extraction for radiocarbon dating. Nature.

[8] Piotrowska N., Goslar T. (2002). Preparation of bone samples in the Gliwice radiocarbon laboratory for AMS radiocarbon dating. Isot. Environ. Health Stud..

[9] Bronk Ramsey C., Higham T., Bowles A., Hedges R. (2004). Improvements to the pretreatment of bone at Oxford. Radiocarbon.

[10] Czernik J., Goslar T. (2001). Preparation of graphite targets in the Gliwice radiocarbon laboratory for AMS ^14^C dating. Radiocarbon..

[11] Goslar T., Czernik J., Goslar E. (2004). Low-energy ^14^C AMS in Poznań radiocarbon laboratory, Poland. Nucl. Instrum. Methods Phys. Res. Sect. B Beam Interact. Mater. At..

[12] Stuiver M., Polach H.A. (1977). Discussion: reporting of ^14^C data. Radiocarbon.

[13] Bronk Ramsey C. (2009). Bayesian analysis of radiocarbon dates. Radiocarbon.

[14] Reimer P.J., Austin W.E.N., Bard E., Bayliss A., Blackwell P.G., Bronk Ramsey C (2020). The IntCal20 northern hemisphere radiocarbon age calibration curve (0–55 cal kBP). Radiocarbon.

[15] Tsutaya T., Yoneda M. (2015). Reconstruction of breastfeeding and weaning practices using stable isotope and trace element analyses: a review. Am. J. Phys. Anthropol..

